# Dual effects of radiation bystander signaling in urothelial cancer: purinergic-activation of apoptosis attenuates survival of urothelial cancer and normal urothelial cells

**DOI:** 10.18632/oncotarget.21995

**Published:** 2017-10-24

**Authors:** Malgorzata A. Bill, Kirtiman Srivastava, Conor Breen, Karl T. Butterworth, Stephen J. McMahon, Kevin M. Prise, Karen D. McCloskey

**Affiliations:** ^1^ Centre for Cancer Research and Cell Biology, School of Medicine, Dentistry and Biomedical Sciences, Queen’s University Belfast, Belfast, BT9 7AE, Northern Ireland, UK

**Keywords:** urothelial cancer, radiation, bystander response, apoptosis, purinergic signalling

## Abstract

Radiation therapy (RT) delivers tumour kill, directly and often via bystander mechanisms. Bladder toxicity is a dose limiting constraint in pelvic RT, manifested as radiation cystitis and urinary symptoms. We aimed to investigate the impact of radiation-induced bystander signaling on normal/cancer urothelial cells. Human urothelial cancer cells T24, HT1376 and normal urothelial cells HUC, SV-HUC were used. Cells were irradiated and studied directly, or conditioned medium from irradiated cells (CM) was transferred to naïve, cells. T24 or SV-HUC cells in the shielded half of irradiated flasks had increased numbers of DNA damage foci vs non-irradiated cells. A physical barrier blocked this response, indicating release of transmitters from irradiated cells. Clonogenic survival of shielded T24 or SV-HUC was also reduced; a physical barrier prevented this phenomenon. CM-transfer increased pro-apoptotic caspase-3 activity, increased cleaved caspase-3 and cleaved PARP expression and reduced survival protein XIAP expression. This effect was mimicked by ATP. ATP or CM evoked suramin-sensitive Ca^2+^-signals. Irradiation increased [ATP] in CM from T24. The CM-inhibitory effect on T24 clonogenic survival was blocked by apyrase, or mimicked by ATP. We conclude that radiation-induced bystander signaling enhances urothelial cancer cell killing via activation of purinergic pro-apoptotic pathways. This benefit is accompanied by normal urothelial damage indicating RT bladder toxicity is also bystander-mediated.

## INTRODUCTION

The goal of radiation therapy (RT) is to maximize the probability of tumour control whilst minimizing damage to surrounding normal tissue. RT for bladder, prostate and cervical pelvic malignancies is associated with radiation-induced bladder toxicity (RIBT) which typically manifests as lower urinary tract symptoms [1–4] due to unavoidable dose delivered to neighbouring normal tissues. Generation of reactive oxygen species and reactive nitrogen species interact with DNA, resulting in damaged lesions including lethal double stranded breaks (DSB) which underpins RT cellular damage. Many cells repair DNA damage through homologous recombination or non-homologous end joining to maintain genome integrity while others, including tumour cells lack effective repair mechanisms and undergo cell death.

Radiation-induced bystander effects describe biological phenomena where non-irradiated cells respond to irradiated neighbouring cells [5]. Therefore, the cellular radiation response incorporates both direct effects and indirect bystander effects leading to genetic instability, diminished survival, apoptosis and necrosis. Bystander mechanisms include intercellular communication via gap junctions and release of transmitters into the extracellular space (or culture media) which then act in an autocrine and paracrine manner. Radiation bystander effects have been described in normal bladder explants correlated with aberrant urothelial outgrowth [6–9] which may be a protective response to urothelial loss that occurs during RT [10], associated with irritative radiation cystitis [11, 12]. In the context of urothelial cancer, studies using the bladder transitional cancer cell line, EJ138, have demonstrated decreased clonogenic cell survival after transfer of medium from irradiated cells, an effect which saturated at 2Gy, consistent with a bystander response [13–15].

The present study tested the hypothesis that ‘radiation bystander signaling occurs in urothelial cancer cells’ and compared the radiobiological response mechanisms with normal urothelial cells. Investigations of DNA damage, clonogenic cell survival and the underlying pathways showed that radiation-induced bystander signaling via purinergic pathways attenuates urothelial cancer cell survival with similar effects on normal urothelial cells which would explain lesions leading to radiation cystitis and RIBT.

## RESULTS

### Tumour and normal urothelial cells exhibit radiation-bystander effects

**Figure 1:**
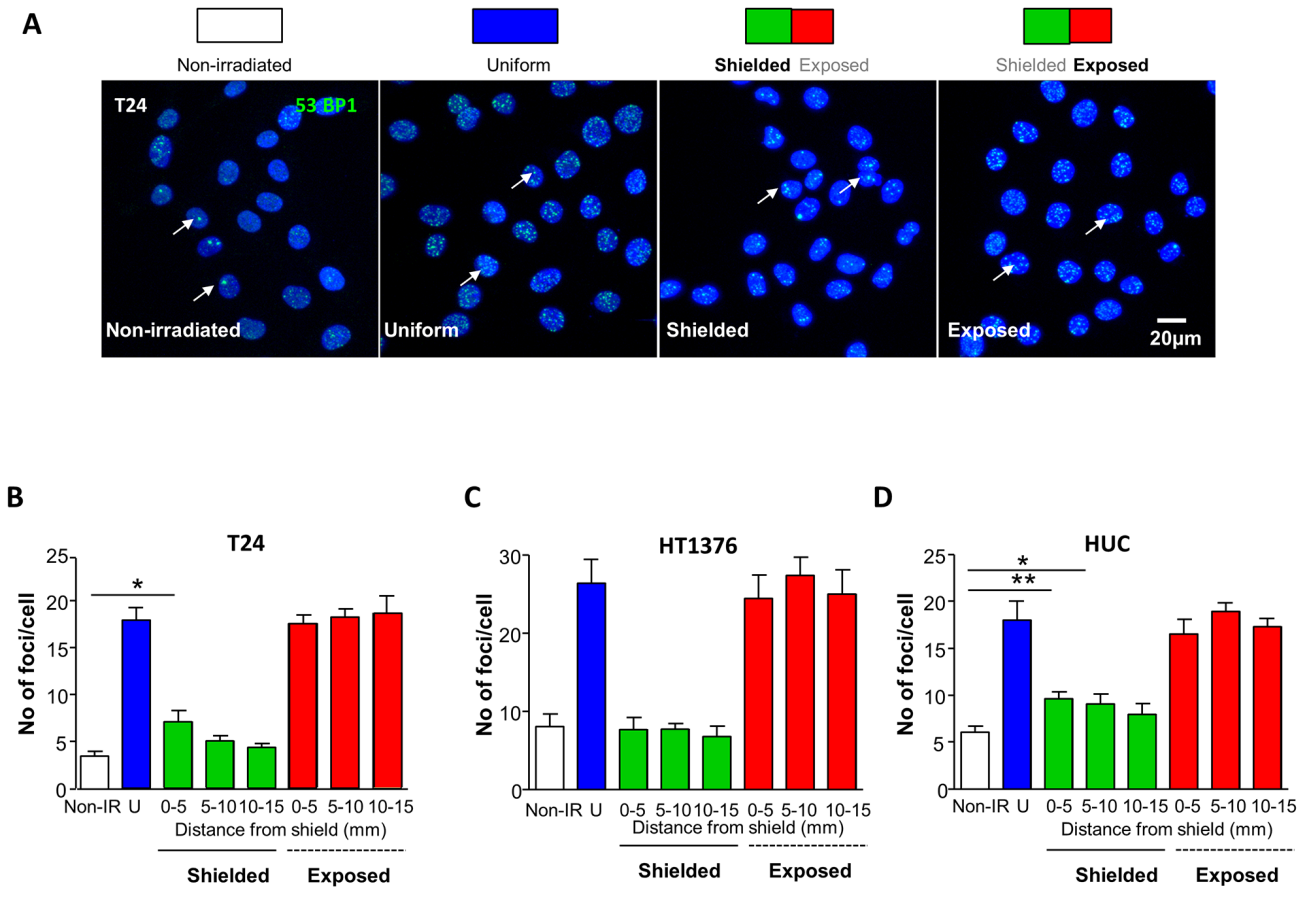
Bystander signaling evokes DNA damage foci formation in urothelial cancer and normal urothelial cells **(A)** Schematic representation of experimental conditions including non-irradiated, uniformly-irradiated and shielding protocols where 50% of the slide was exposed and 50% shielded with MCP alloy. Cells were fixed and processed for 53BP1-immunofluorescence, one hour following irradiation. Micrographs of urothelial cancer T24 cells labelled with anti-53BP1 (green) to label nuclear DNA double stand break foci and DAPI to counterstain nuclei are shown for each experimental condition. **(B)** Summary graph of mean foci/cell in T24 cells (N=3), measured from confocal z-stacks in Volocity software. There were significantly more foci in shielded cells 0.5mm from the shield than in non-irradiated controls, indicative of a bystander effect (p=0.019). **(C)** Summary data for HT1376 cancer cells (N=3) where there was no difference in the mean foci/cell between non-irradiated controls and shielded cells. **(D)** Summary data for normal urothelial HUC (N=3). Mean foci/cell was significantly greater in shielded cells in the 0-5mm (p=0.005) and 5-10mm (p=0.04) analysis regions from the shield. * denotes p<0.05, ** denotes p<0.01

Nuclear foci comprising 53BP1 proteins [16] formed around DNA DSB are used to quantify DNA damage. Automatic foci-scoring of confocal z-stacks was used to minimise any investigator-related unconscious bias [17]. Foci were measured in cells within non-irradiated, uniformly irradiated and shielded/exposed flasks (Figure 1A, Supplementary Figure 1). Cells were fixed and processed for 53BP1-immunofluorescence one hour following irradiation exposure. X-ray irradiation (1Gy) increased the mean number of 53BP1 foci in uniformly-irradiated (17.9±1.3) vs non-irradiated T24 (3.6±0.4, p<0.0001, Figure 1B). Foci in exposed regions were similar to cells in uniformly-irradiated chambers (p>0.05). Interestingly, the number of foci in shielded regions (up to 5mm from the analysis region at the flask centre) was larger than in non-irradiated cells (7.1±1.2; p=0.019) consistent with bystander signaling as the dose in this region was only 0.03 Gy. Although not statistically significant, the data suggest that foci in the adjacent shielded region (5-10mm) also increased slightly (5.1±0.6) vs control (p=0.07).

HT1376 urothelial carcinoma cells, had the expected increased foci in uniformly-irradiated vs non-irradiated flasks (Figure 1C), however, similar numbers of foci per nucleus in shielded regions vs non-irradiated cells were observed (p>0.05) indicating absence of a bystander response.

Normal HUC (Figure 1D) also had increased numbers of foci per nucleus in uniformly-irradiated (18±2.0) vs non-irradiated (6.1±0.6, p=0.0002) flasks. Similar to T24, the number of foci in HUC from uniformly-irradiated chambers was similar to exposed regions (p>0.05). The number of foci per nucleus in shielded sections were larger than in non-irradiated chambers (0-5mm 9.6±0.8, p=0.005; 5-10mm 9.1±1.1, p=0.04) consistent with bystander signalling.

### Physical barrier abolishes bystander DNA DSB foci

**Figure 2:**
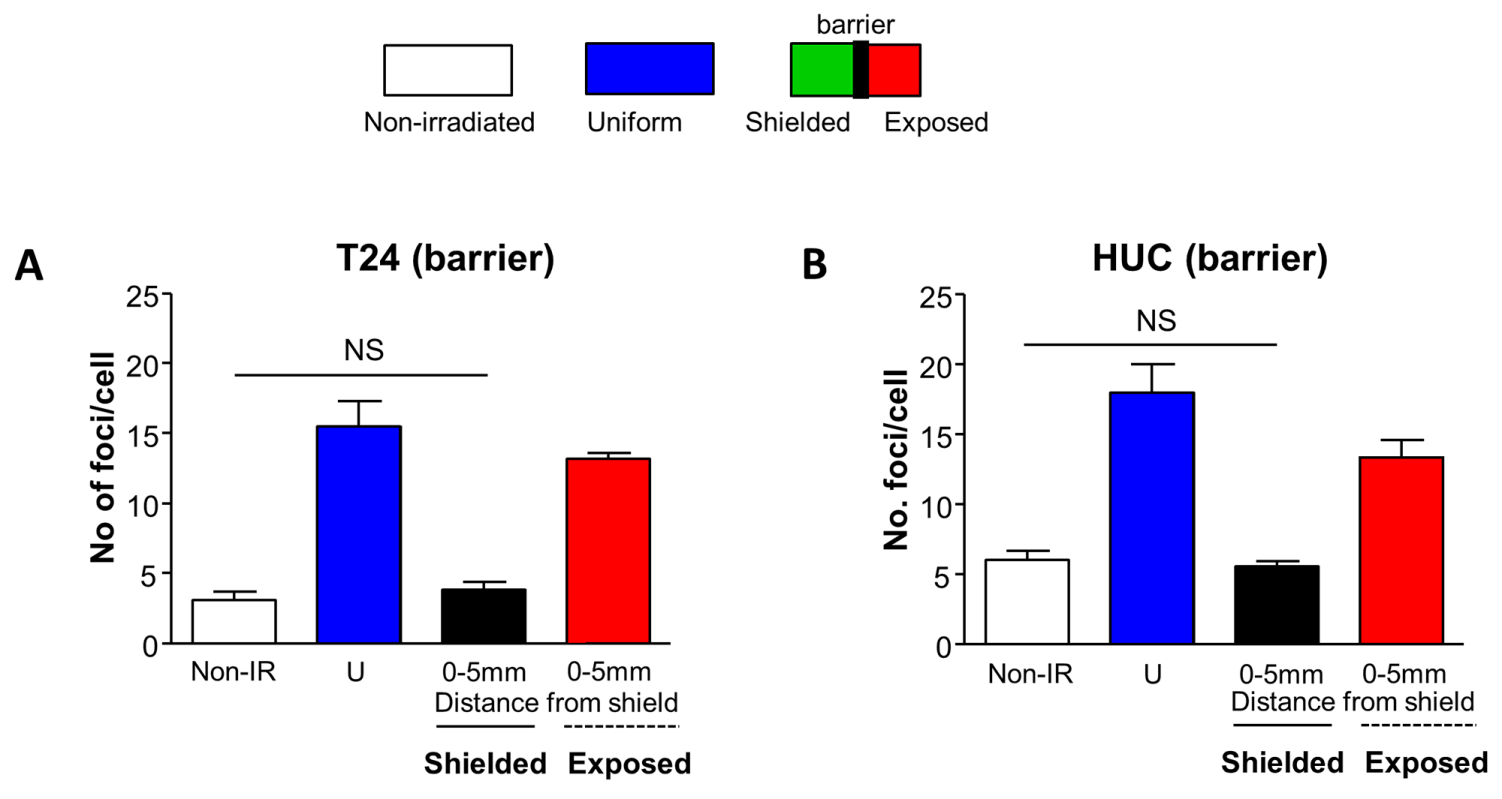
Bystander DNA damage foci are prevented by a physical barrier Schematic representation of experiments similar to those presented in Figure 1, with a physical barrier between shielded and exposed sections. Cells were fixed and processed for 53BP1-immunofluorescence one hour following irradiation. **(A)** Inclusion of a physical barrier prevented formation of bystander DNA damage foci in shielded T24 cells (N=3). **(B)** Similarly, the physical barrier prevented formation of bystander DNA damage foci in shielded HUC cells (N=3)

To investigate whether intercellular communication through transmitter release from irradiated cells contributed to bystander DSB in shielded regions, communication between shielded and exposed regions was physically inhibited. The bystander effect observed in Figure 1 was abolished in the shielded region for both T24 (Figure 2A) and HUC (Figure 2B) cells when communication was physically inhibited.

### Relative radiosensitivities of urothelial cells and impact of radiation on cell survival

**Figure 3:**
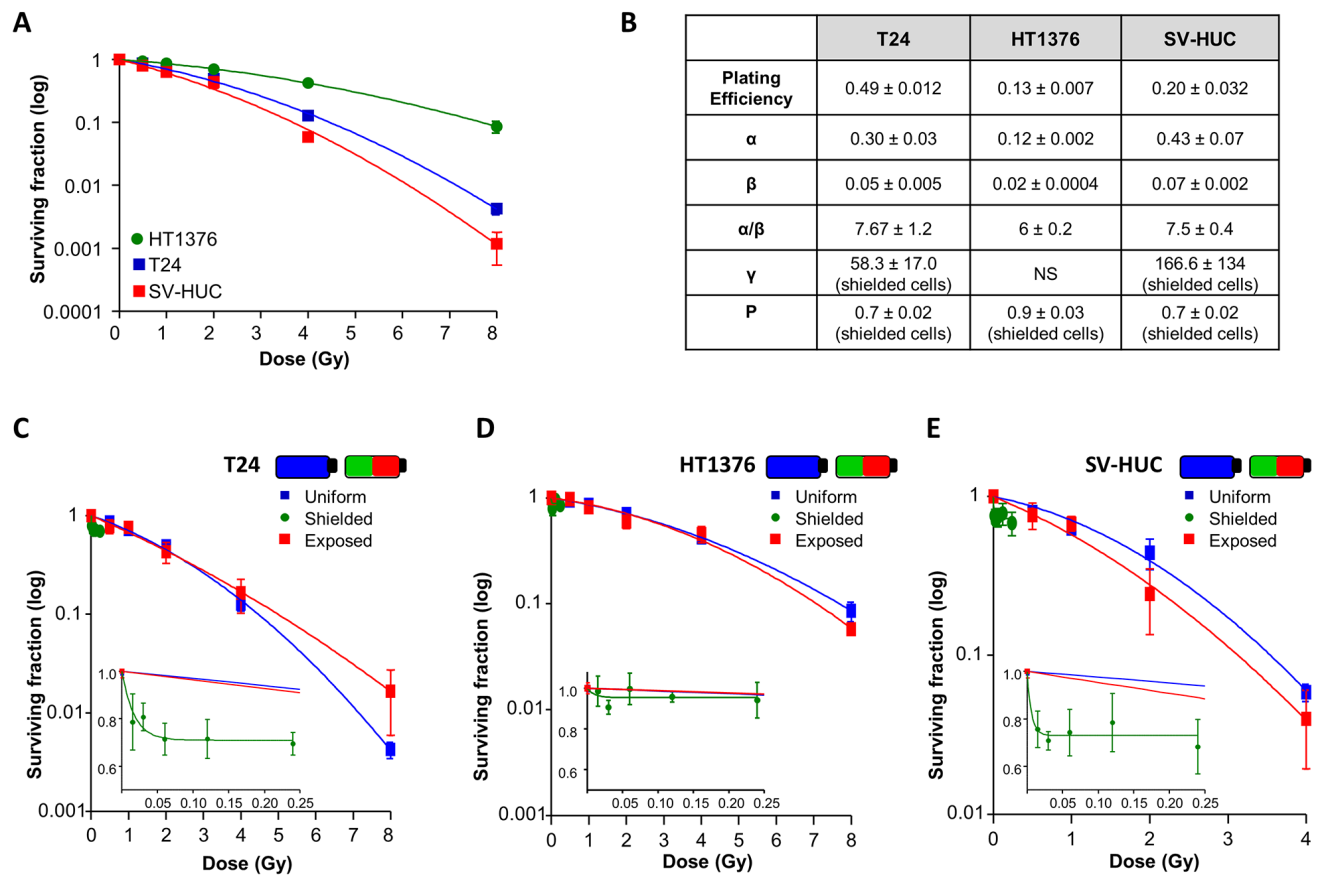
The bystander effect decreases cell survival of T24 and SV-HUC **(A)** Surviving fraction of T24, HT1376 and SV-HUC cells plotted against the radiation dose range 0.5Gy-8Gy (all N=3). HT1376 cells were the most radioresistant and normal SV-HUC were most radiosensitive. **(B)** Summary data from linear quadratic (LQ) modelling of survival curves for the 3 cell lines in uniformly-irradiated, shielded and exposed conditions shown in panels **(C-E)** α represents a single hit killing mechanism e.g. non-reparable DNA double stand breaks, greatest in SV-HUC whereas β represents multiple hit processes. The α/β ratio represents the dose at which both processes play an equal role e.g. the higher the ratio, the less repair will be occurring. The γ value shows the response rate where a higher value describes rapid response. Here, SV-HUC have the highest γ therefore the highest fraction of cells responding at the highest rate. (C) Survival curve for T24 under the 3 conditions (N=3). At 8Gy, cells in the exposed section had a survival advantage compared with uniformly-irradiated cells indicative of a protective bystander effect. Inset shows decreased survival of shielded cells plotted against the calculated scattered dose during each exposure and saturates around 70% survival at a scattered dose of 0.05Gy. (D) Similar curves for HT1376 (N=3) showing absence of a bystander effect on survival with little effect on shielded cells. (E) SV-HUC cells had a marked bystander response with decreased survival in shielded cells (N=3) that saturated at 60-80% survival at a scattered dose of 0.24Gy.

Relative cellular radiosensitivities were investigated in clonogenic survival assays over 0.5Gy-8Gy dose-range. Summary data of the surviving fractions (Figure 3A) demonstrates HT1376 cells to be more radioresistant and normal SV-HUC and T24 to be more radiosensitive.

Cell survival curves were fitted with Linear Quadratic (LQ) modelling (Figure 3B) which confirmed SV-HUC as the most radiosensitive (α=0.43±0.07) particularly >2Gy. T24 were also radiosensitive (α=0.30±0.03) >2Gy compared with the relatively radioresistant HT1376 (α=0.12±0.002), consistent with [18]. HUC radiosensitivity could not be assessed due to inability of these cells to form colonies.

Cells were irradiated in T25 flasks either uniformly or in shielded/exposed flasks (Supplementary Figure 2). Summary survival curves (Figure 3C-3E) were determined for each condition where the surviving fraction is plotted against dose, which for shielded regions is the calculated scattered dose, shown in the insets on an expanded scale (Figure 3B). As bystander responses typically saturate and do not affect the whole cell population, the non-responding cell population is defined as P (bystander limit), and the rate of bystander response is characterised by γ. The shielded region survival is therefore SF=P+(1-P)*exp(-γ*D), where D is the dose delivered to the exposed part. The initial slope of this curve is (1-P)*γ, comparable to α in the LQ [19].

T24 in exposed sections had increased survival (8Gy) vs uniformly-irradiated cells, indicative of a bystander-mediated advantage. Similar analysis of cells in shielded sections showed decreased survival (P=0.7±0.02, γ=58.3±17, (1-P)*γ=17.5), an effect which saturated at a scattered dose of 0.05Gy at 70% survival.

The exposed section survival of SV-HUC cells (α=0.43±0.07) was reduced compared with uniformly-irradiated (α=0.47±0.03) at 4Gy. Cell survival in shielded regions was decreased compared with LQ prediction and reached 60-80% at a scattered dose of 0.24Gy (P=0.7±0.02, γ=166.6±134, (1-P)*γ=44.9). The radioresistant HT1376 in exposed sections were similar to uniformly-irradiated. Likewise, the survival curve for the shielded region matched uniformly-irradiated and exposed sections showing a lack of bystander response.

### Bystander cell survival effect was prevented by a physical barrier

To assess the involvement of intercellular communication, cells were seeded in 6-well plates, where communication between shielded and exposed cells was physically inhibited. Cell survival curves for T24 or SV-HUC in exposed sections (Supplementary Figure 3) were similar to uniformly irradiated flasks; moreover, cell survival curves for shielded sections was close to that predicted by the LQ model. These results indicate a direct role for cell communication in bystander survival responses of T24 and SV-HUC.

### Irradiation evokes ATP release from T24 cells

**Figure 4:**
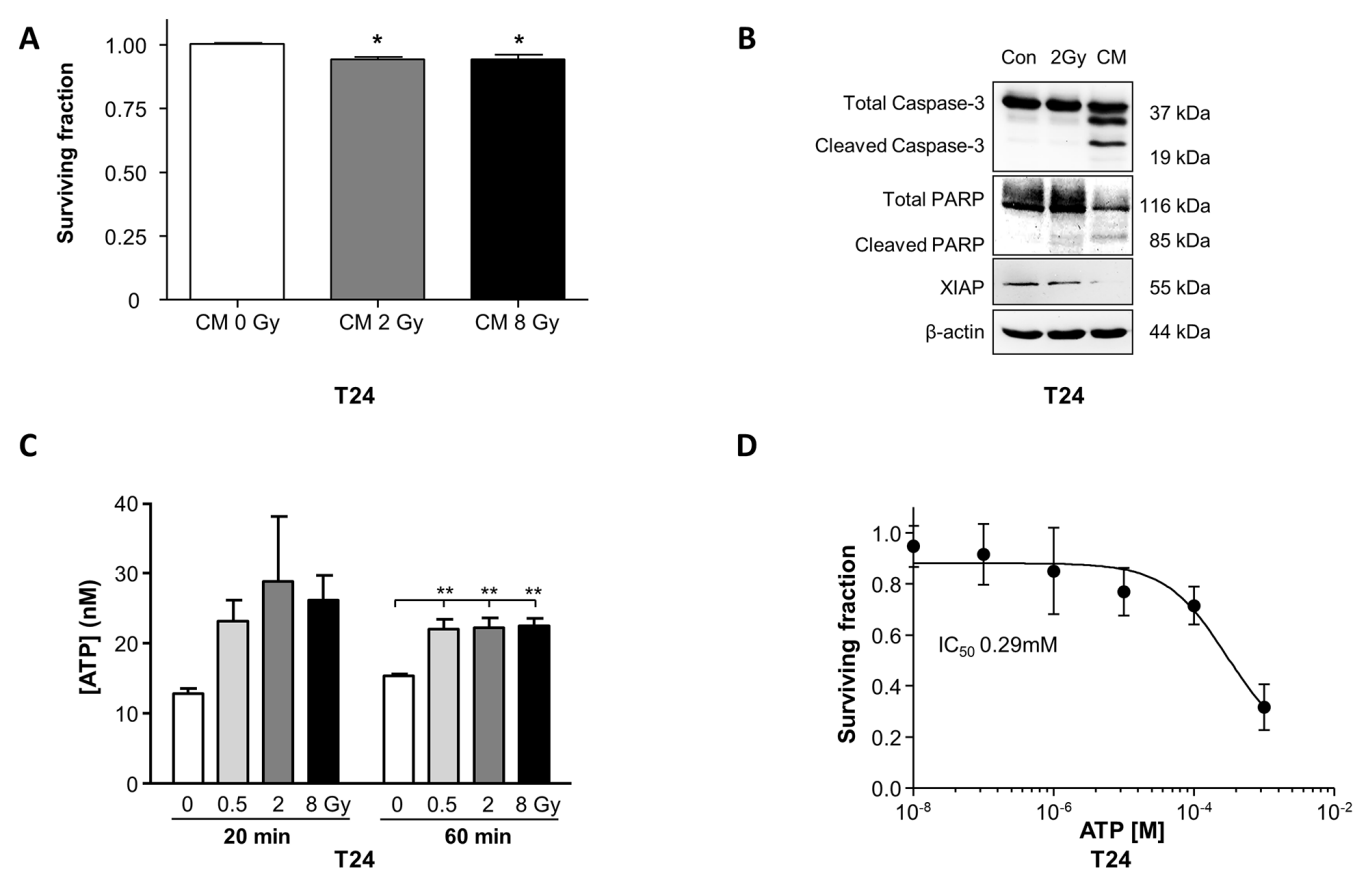
Radiation-induced bystander effects diminish cell survival of T24 cells **(A)** Application of conditioned medium (CM) from irradiated (2Gy, 8Gy) T24 to non-irradiated cells significantly decreased clonogenic cell survival (N=3, p<0.05). Control experiments used CM from non-irradiated cells (Gy). **(B)** T24 cells that were directly irradiated (2Gy) or received CM from directly-irradiated cells (2Gy) were processed for Western blotting. CM evoked expression of cleaved caspase-3, cleaved PARP and reduced XIAP expression, consistent with activation of pro-apoptotic signaling (represents N=3). **(C)** ATP concentration was enhanced in CM obtained from cells 20 min post-irradiation at 0.5Gy, 2Gy and 8Gy and was significantly increased in CM obtained from cells irradiated at 0.5Gy, 2Gy and 8Gy (N=3, p<0.01) one hour post-irradiation. ARL67156 (50μM), an ectoATPase inhibitor, was included in these experiments to prevent ATP hydrolysis. **(D)** T24 clonogenic cell survival was concentration-dependently decreased by exogenous ATP treatment with an IC_50_ of 0.29mM (N=3).

CM transfer from irradiated cells to non-irradiated cells is a common protocol to investigate bystander effects. Controls were performed where CM was transferred from non-irradiated cells to naïve cells. CM significantly decreased T24 clonogenic survival (p<0.05, Figure 4A) therefore Western blots of pro-apoptotic pathways were performed on T24 that were directly-irradiated or received CM. In both conditions, cells expressed increased pro-apoptotic proteins; cleaved caspase-3, cleaved PARP and reduced XIAP (inhibitor of apoptosis proteins). ATP release after irradiation has been reported in other cells [20]; in T24, ATP was enhanced in CM at 20 min post-irradiation and was significantly enhanced in CM 1h post-irradiation, analysed with 1-way ANOVA (P=0.0065) (Figure 4C) and with post-hoc Dunnett’s test from 15.3±0.3nM (non-irradiated) to 21.9±1.5nM (0.5Gy, P<0.01), 22.1±1.5nM (8Gy P<0.01) and 22.5±1.1nM (8Gy P<0.01). When the two timepoints were combined for 2-way ANOVA, radiation exposure had a significant effect (P=0.03) whereas time had no overall effect (P>0.05). These experiments were carried out in the presence of the ectoATPase inhibitor, ARL67156 to prevent ATP hydrolysis.

### ATP modulates bystander signalling in T24 cells

**Figure 5:**
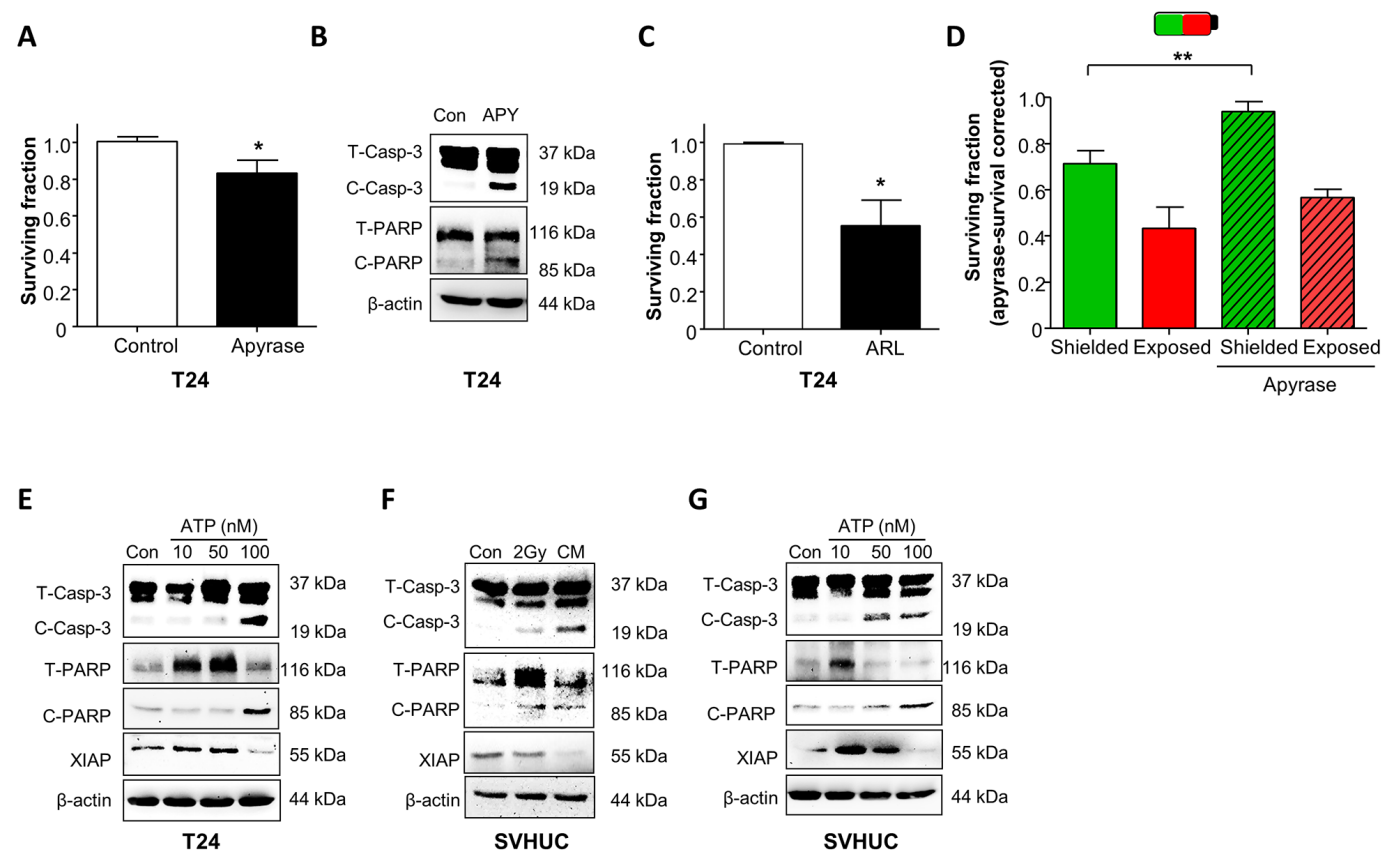
Radiation-induced bystander effects in urothelial cancer and normal urothelial cells is mediated by purinergic-signalling **(A)** The ATP diphosphohydrolase, apyrase (10U/ml) significantly decreased T24 cell survival (N=3, p<0.05). **(B)** Apyrase (10U/ml) treatment increased expression of cleaved caspase-3 (C-Casp-3) and cleaved PARP (C-PARP). Total caspase-3 and total PARP are abbreviated to T-CASP-3 and T-PARP-3 respectively (represents N=3). **(C)** ARL67156 (100μM), an ectoATPase inhibitor decreased T24 cell survival (N=3, p<0.05). **(D)** In shielding protocols, the decreased survival of shielded cells was rescued by apyrase treatment N=3, p<0.01). Survival was corrected for apyrase effects on non-irradiated cells. **(E)** ATP activated pro-apoptotic signaling in a concentration-dependent manner with increased expression of cleaved caspase-3, cleaved PARP and decreased XIAP expression (N=3). **(F)** Similar experiments were performed on normal SV-HUC cells; directly irradiated cells had upregulation of cleaved caspase-3 and cleaved PARP. CM also increased expression of these proteins and also inhibited expression of XIAP. **(G)** The CM bystander effect on normal SV-HUC was mimicked by ATP treatment across a concentration range.

To independently test whether ATP within CM decreased cell survival, exogenous ATP was applied to T24 (10nM-1mM) and dose-dependently decreased survival, IC_50_ 0.29mM (Figure 4D). Interestingly, T24 require basal ATP for physiological survival and either excess (Figures 4D and 5C) or decreased ATP levels (Figure 5A) reduced survival. The ATP diphosphohydrolase, apyrase (breaks down ATP, 10U/ml) decreased the surviving fraction to 0.83±0.05 (p<0.05, Figure 5A), confirmed by the presence of cleaved caspase-3 and cleaved PARP, consistent with activation of apoptosis (Figure 5B). Enhancement of basal ATP with the ectoATPase inhibitor ARL67156 (100μM, prevents ATP breakdown) reduced the surviving fraction to 0.58±0.08 (p<0.05, N=3, Figure 5C) demonstrating the importance of extracellular ATP homeostasis.

In further experiments, flasks were pre-treated with apyrase (30min) and irradiated in exposed/shielded sections (2Gy). Non-irradiated controls, with/without apyrase were included. As apyrase decreases T24 survival (Figure 5A), survival responses in shielded/exposed cells were calculated relative to apyrase-treated, non-irradiated controls. Apyrase rescued the surviving fraction in shielded sections (0.75±0.03 to 0.94±0.06, N=3, p<0.01), consistent with ATP-modulated bystander signalling. Confirmation that ATP activated pro-apoptotic signaling pathways, mimicking the CM effect is presented in Figure 5E, where ATP dose-dependently increased cleaved caspase-3, cleaved PARP and decreased XIAP expression.

### Bystander signaling or ATP activates pro-apoptotic pathways in normal urothelial cells

We then investigated whether similar pathways occur in normal urothelial cells. In SV-HUC (Figure 5F), direct irradiation enhanced cleaved caspase-3 and cleaved PARP expression; this was more apparent in CM-treated, non-irradiated cells. Interestingly, while direct irradiation did not impact pro-survival XIAP expression, CM blocked XIAP expression, showing that bystander signaling further promoted apoptosis. These findings were mimicked by ATP (Figure 5G) confirming ATP-mediated toxicity in SV-HUC.

### Purinergic-mediated bystander signaling in T24 cells is Ca^2+^-dependent

**Figure 6:**
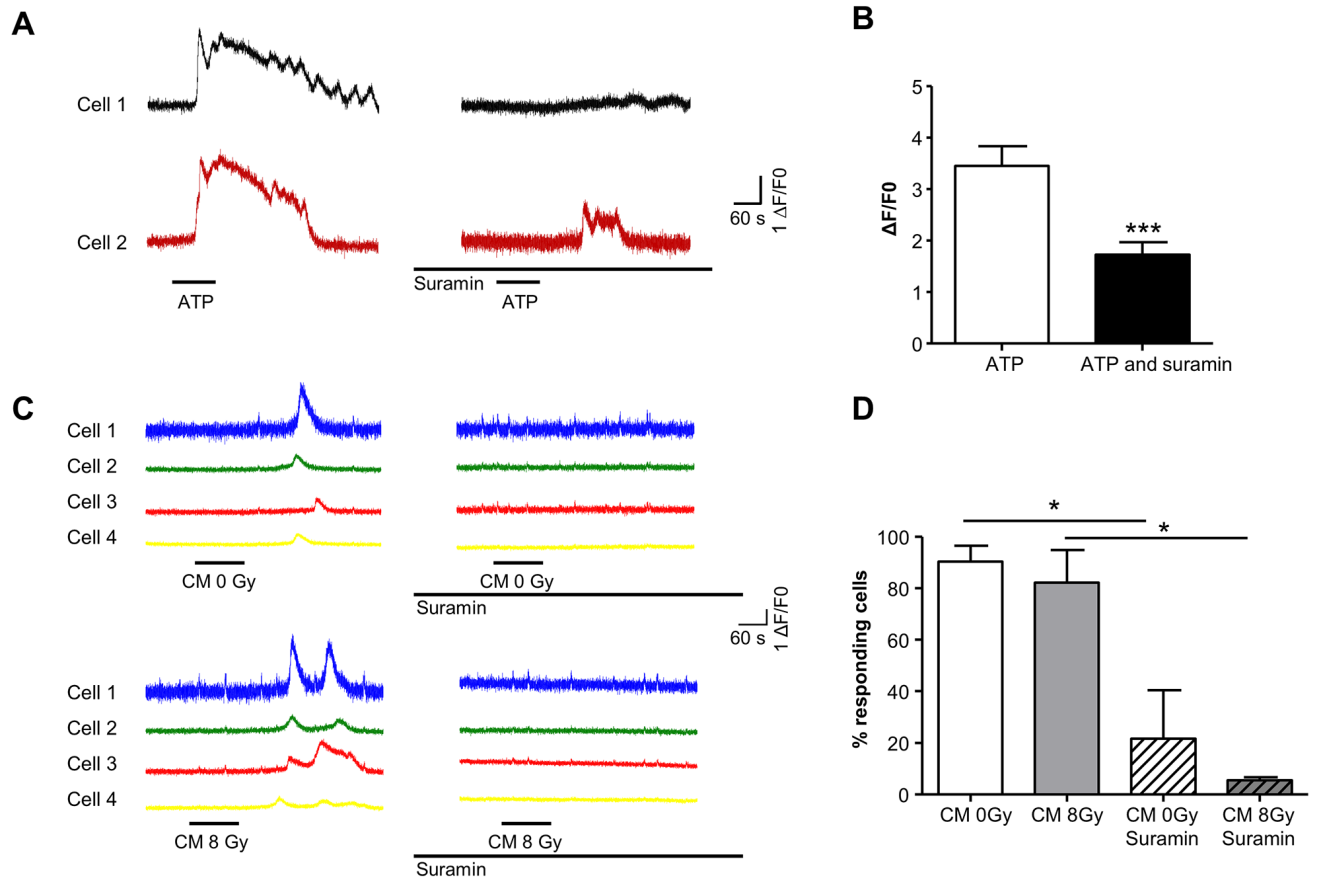
Purinergic receptor mediated bystander effects evoke Ca^2+^-transients in T24 cells **(A)** T24 cells, loaded with the Ca^2+^-indicator, fluo-4AM were treated with ATP (10^-5^M) as indicated by the horizontal bars. Responses from 2 cells within the recording dish are presented and show transient increases in intracellular Ca^2+^. After washout and incubation with the pan-purinergic receptor blocker, suramin (10^-4^M), ATP treatment was repeated and responses were markedly reduced or blocked. **(B)** Summary data for 51 cells (N=3) is presented in the bar chart where suramin significantly reduced the amplitude of ATP-evoked responses (p<0.001). **(C)** In another experimental series, T24 cells were treated with conditioned medium (CM) from non-irradiated (0 Gy) followed by a 10 minute wash and then CM from irradiated cells (8Gy). Four cells are shown from the recording dish. The cells responded to CM (0 Gy and 8 Gy) with Ca^2+^-transients. CM was then washed out and cells treated with suramin (10^-4^M) for 30 minutes before application of CM in the presence of suramin which blocked the responses. **(D)** The summary bar chart shows the percentage of cells responding to CM in the absence of suramin and the marked reduction of responding cells after suramin treatment (N=3, n=72).

Exogenous application of ATP (10^-5^M) to fluo-4AM loaded T24 cells evoked increases in fluorescence, indicating increased intracellular [Ca^2+^]. This concentration of ATP was found (above) to significantly decrease T24 clonogenic cell survival. ATP-evoked responses were repeatable after a 10 min washout. After control responses were obtained (Figure 6A), cells were washed and exposed to the pan-purinergic receptor blocker, suramin for 30 min (10^-4^M). Subsequent application of ATP in the presence of suramin evoked significantly smaller responses (ΔF/F_0_ from 3.5±0.4, to 1.7±0.2, N=3, n=51 cells; p<0.001, Figure 6B).

To examine whether conditioned medium (CM) from irradiated cells could evoke Ca^2+^-transients, CM from non-irradiated (0 Gy) or irradiated (8Gy, 1h after irradiation) cells was applied. Intensity-time plots for Ca^2+^-transients evoked by CM from control (0Gy) and 8Gy experiments are shown in Figure 6C. Both 0Gy CM and 8Gy CM evoked Ca^2+^ transients in recipient cells, consistent with the finding that ATP is released from non-irradiated cells and at a larger concentration from irradiated cells (see above). To determine whether the CM-evoked responses were mediated by purinergic signaling, CM from cells from control 0 Gy or 8 Gy irradiated cells was applied (applications were separated by a 10 minute wash) and subsequently, cells were incubated with suramin (10^-4^M) for 30 minutes. Suramin reduced the percentage of cells responding to both 0Gy from 90.4±6.2% to 21.5 ± 18.9% and 8Gy from 82.3±12.6% to 5.5±1% (8Gy) (N=3, n=72, Figure 6D).

## DISCUSSION

These novel findings demonstrate the impact of radiation-induced bystander signaling in cell death of normal and cancer urothelial cells. Radiation-induced bystander signaling is potentially both advantageous through maximizing tumour cell death and undesirable through damage to normal urothelial cells, contributing to RIBT.

The typical tumour response to radiation is largely determined by radiosensitivity of cancer cells [21]. Here, normal SV-HUC were most radiosensitive, and HT1376 urothelial cancer cells were most radioresistant, consistent with [18]. Normal urothelial cell radiosensitivity is important clinically as the urothelium is important in post-irradiation bladder pathogenesis [22]. Divergence of the SV-HUC survival curve from urothelial cancer cells >2Gy represents the typical radiation response of increased radiosensitivity in normal cells. This rationale underpins standard clinical 2Gy dose fractions as an optimal balance between cancer cell kill and normal cell protection [4].

LQ modelling of cell survival demonstrates total lethal lesions comprising those produced by: (1) a single hit (linearly related to dose) and (2) two hits (quadratically related to dose) [23]. The similar α/β ratio found here between T24, HT1376 and SV-HUC cells shows that differences in radiosensitivity were not due to different *types* of damage, produced by multiple hits, but may potentially represent *deregulated* cell cycle control in the cancer cell lines (particularly HT1376) however, this was not directly investigated in the present study. Here, the bystander effect correlated with radiosensitivity and was absent in the most resistant cell line. Cells are typically most radiosensitive in M and G2 phases while most are radioresistant in S phase. For cells with a long cycle e.g. HT1376 (doubling time 36h vs 19h T24), there is also increased resistance in early G1. Correlation of radiosensitivity and length of the cell cycle has been shown in cell lines [24] and lymphocytes [25]. In other studies, irradiated regions of human urothelial explants using microbeams correlated with differentiation and proliferation status resulting in outgrowth of neighbouring non-irradiated regions [6, 7].

The shielding vs exposed experimental design models Intensity-Modulated RT (IMRT) where cells are irradiated close to neighbouring non-irradiated cells and steep dose-gradients exist. For cells with bystander effects (T24 and SV-HUC), survival in the shielded region was lower than that predicted from the scattered dose. Bystander effects were absent in radioresistant HT1376 cells showing correlation between radiosensitivity and bystander signaling, consistent with [19].

T24 cancer cells in exposed regions had increased survival at high doses, vs uniformly-irradiated, suggesting a counteracting effect to the decreased survival of shielded cells; a similar phenomenon has been reported for other cell lines [19, 26, 27]. SV-HUC showed opposite effects, where exposed cells had decreased survival vs uniformly-irradiated regions. In SV-HUC, there might be greater damage in IMRT type regimens even at therapeutically relevant 2Gy fractions.

T24, HT1376 and HUC had significantly increased 53BP1 foci, one hour after irradiation. Interestingly, in shielding experiments, increased 53BP1 foci occurred in shielded T24 (0-5mm) and SV-HUC (0-10mm) from the edge of the shield. A similar phenomenon has been reported for prostate cancer DU145 cells [19] similar to the findings here, where increased DNA damage foci within the region closest to the border of the shielding is consistent with diffusion of transmitters from cells in exposed sections. The prevention of bystander DNA foci in shielded cells by a physical barrier supports this hypothesis. Interestingly, consistent with absence of a bystander cell survival effect in the radioresistant HT1376 cells, increased foci per nucleus did not occur in the shielded region.

The finding that radiation enhanced ATP release from T24 cells indicated that ATP within CM might be a candidate for mediating the bystander effect. This was confirmed by a dose-dependent reduction of cell survival by ATP and its activation of pro-apoptotic signaling pathways. Activation of executioner caspase-3 by proteolytic cleavage of its pro-enzyme is an apoptosis hallmark. Active caspase-3 cleaves and impairs the DNA-repair enzyme poly-ADP ribose polymerase (PARP), which compounds DNA damage directing cells towards apoptosis [28]. T24 rely on basal ATP for survival as promotion or prevention of ATP breakdown by apyrase or ARL67156 respectively reduced survival, indicative of ATP homeostasis. The enhanced release of ATP by radiation therefore unsurprisingly leads to apoptosis and associated signaling pathways. Rescue of survival reduction in shielded cells from bystander signaling by apyrase further supports the role of ATP release from irradiated cells which diminished cell survival in neighbouring cells. Reduction of xenograft urothelial [29] or prostate [30] tumour growth by daily intraperitoneal injections of ATP (mM) has been reported, moreover, inhibition of purinergic receptors also decreases tumour growth [31].

ATP acts as a signaling molecule via purinergic receptors which fall broadly into two families, G-protein coupled receptors (P2Y) and ATP-receptor activated membrane ion channels (P2X). ATP signal transduction involves intracellular Ca^2+^-signaling (transient increases in intracellular Ca^2+^ concentration) which occur rapidly i.e. within seconds-minutes of exposure, subsequently activating downstream pathways. We found that T24 cells release ATP and also respond to exogenous ATP with Ca^2+^-transients that were inhibited by purinergic receptor blockade. It was beyond the scope of the present study to carry out a full characterization of the purinergic receptors mediating ATP effects, moreover, the expression of purinergic receptors on T24 cells has not yet been reported. HT1376 cells express (P2X_4,5,7_ and P2Y_1,2,4,6,11)_) [29], however as they did not exhibit bystander responses in the present study, they were not studied further. The bystander CM Ca^2+^-response in T24 cells was blocked by suramin demonstrating its mediation by purinergic receptors. Interestingly, CM from non-irradiated cells also evoked suramin-sensitive Ca^2+^-responses, consistent with the finding that T24 have basal ATP release and their survival relies on ATP homeostasis. Our findings demonstrate that in T24 urothelial cancer cells, radiation evokes release of ATP which underpins bystander responses of decreased cell survival. Activation of the pro-apoptotic signaling pathway including executioner cleaved caspase 3 and cleaved PARP is consistent with the CM-evoked Ca^2+^-responses as both caspase 3 and PARP are known to be Ca^2+^-dependent [32–34].

**Figure 7:**
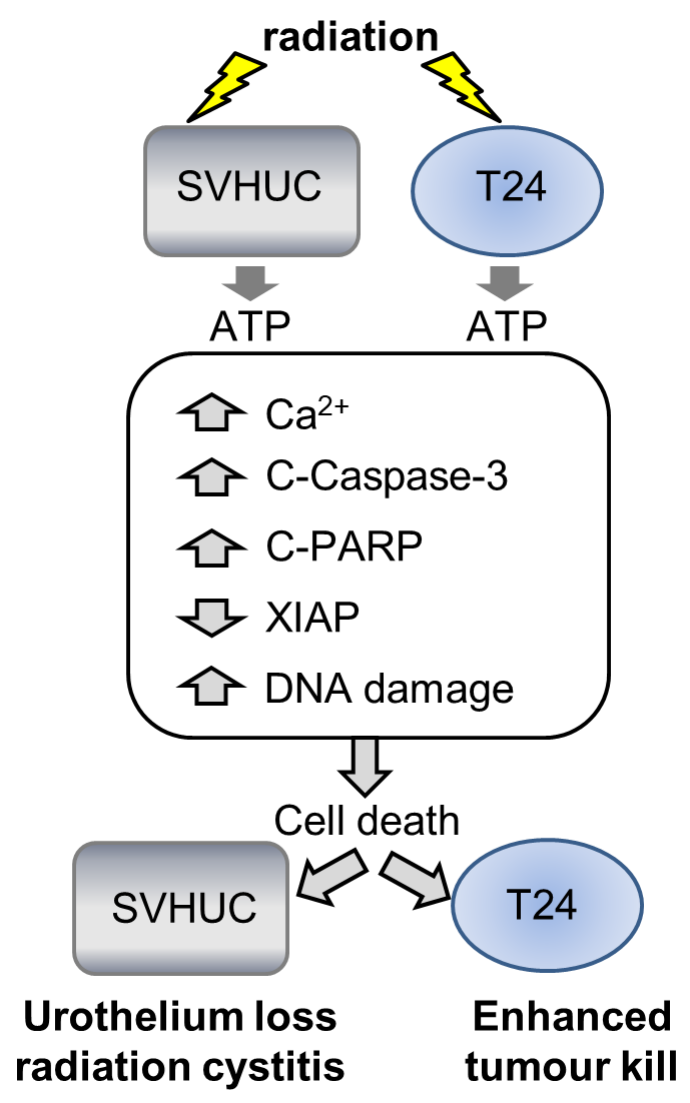
Summary schematic Summary schematic diagram of the radiation-bystander effect that is common to urothelial cancer (T24) cells and normal urothelium (SV-HUC). Directly irradiated cells signal to bystander cells activating pro-apoptotic signaling mechanisms and causing DNA double stand break damage resulting in decreased cell survival. The bystander effect studied here was mediated by ATP release from irradiated cells that acted on bystander cells.

In conclusion, radiation activates bystander signaling in urothelial cancer cells which causes DNA damage, activation of pro-apoptotic pathways and reduced cell survival via ATP-purinergic, Ca^2+^-dependent mechanisms (Figure 7). This advantageous effect is accompanied by similar effects on normal urothelial cells, underpinning radiation-induced bladder toxicity and symptoms of radiation cystitis.

## MATERIALS AND METHODS

### Cell models

Urothelial carcinoma cells T24 (McCoy’s 5A) [35], HT1376 MEM) [36], immortalised normal urothelial cells SV-HUC (F12K) [37], were purchased from ATCC; primary normal HUC (Urothelial Cell Medium) were obtained from ScienCell Research Laboratories. Cells were cultured in their respective medium (parentheses), supplemented with 10% FBS and 1% penicillin-streptomycin.

### Cell irradiation

Radiation was delivered (13.3mA, 225kVp, 2mm Cu filtered, 0.59Gy/min; 0.5-8Gy) in an XRAD225 X-ray cabinet (Precision X-ray Inc. Bradford, USA). For partial irradiations in bystander studies, 50% of T25 flask areas was shielded with a 13.6x10.4x2.1cm^3^ block of MCP96 (MCP Ltd, Wellingborough, Northants, UK). Scattered dose under the shielding was 2-3% of the full dose delivered to the exposed region, determined using GAFCHROMIC® RTQA film (International Specialty products, Wayne, USA) [19, 27].

### Clonogenic cell survival assay

Clonogenic assays were performed in non-irradiated, uniformly-irradiated or 50% shielded T25 flasks (shielded vs exposed) as previously described [19, 27]. 6-well plates were used in experiments where communication was inhibited with a physical barrier. Medium transfer experiments were performed using conditioned medium (CM, 0.2μm filter) from irradiated donor cells and transferred to non-irradiated recipient cells. In controls, conditioned medium refers to medium from non-irradiated cells transferred to naïve cells. After irradiation/CM transfer, flasks/plates were cultured until colonies (>50 cells) formed (8d T24, 12d SV-HUC, 16d HT1376).

### Analysis of clonogenic survival assays in irradiation protocols

Colonies were visually counted; in T25 flasks, a 5mm region at the shield and flask edges was omitted; middle wells of 6-well plates were not analysed. Plating efficiency (PE) was calculated as number of colonies/number of cells and the surviving fraction (SF) as Experimental PE/Control PE. Data was fitted with the linear-quadratic (LQ) model which defines two components of cell killing: αD, proportional to the dose and βD^2^, proportional to dose^2^. The α/β component for uniform irradiation was calculated using the equation SF=exp(-αD-βD^2^). Cell survival data in Figure 3 was fitted using a two-component radiation response model [27] which refers both to the scattered dose in the shielded region and a bystander component triggered by the dose delivered to the exposed region. As bystander responses typically saturate and do not affect the whole cell population, the non-responding cell population is defined as P (bystander limit), and the rate of bystander response is characterised by γ. The shielded region survival is therefore SF=P+(1-P)*exp(-γ*D), where D is the dose delivered to the exposed part. The initial slope of this curve is (1-P)*γ, comparable to α in the LQ.

### DNA damage foci

Cells were seeded in slide-flasks (Nunc, UK) or Millicell EZ slides (Millipore, UK) where communication between shielded and exposed parts was inhibited. Cells were irradiated (1Gy) in uniformly-irradiated and shielded protocols, fixed one hour following irradiation and processed for immunofluorescence with anti-53BPI (Table 1) and DAPI [19, 27]. Gafchromic film placed under each slide flask was used to define the shielded area to be scored, with typically a 5mm exclusion region based on the Gafchromic film signal. Slides were imaged with confocal microscopy (Nikon C1). Laser/gain settings were identical for control and experimental slides. Images were captured as z-stacks. Negative controls were prepared with omission of primary antibody. Foci were scored within 5mm intervals on each side of the shielding [19] in 50 nuclei/sample using automation in Volocity software (Perkin Elmer).

**Table 1:** List of antibodies

Antibody	Company	Product number	Host species	Dilution
53BP1 (IF)	Novus Biologicals	NB100304	Rabbit	1:1,000
anti-rabbit Alexa Fluor 488 (IF)	ThermoFisher	A-11034	Goat	1:500
total caspase-3 (WB)	Cell signalling	9665S	Rabbit	1:1000
cleaved caspase-3 (WB)	Cell signalling	9664S	Rabbit	1:1000
PARP (WB)	Cell signalling	9532S	Rabbit	1:1000
cleaved-PARP (WB)	Cell signaling	9541S	Rabbit	1:500
β-actin (WB)	Sigma-Aldrich	A5441	Mouse	1:20,000
anti-rabbit IgG HRP (WB)	Cell signalling	7074S	Rabbit	1:6000
anti-mouse IgG HRP (WB)	Cell signalling	7076S	Mouse	1:6000

IF: immunofluorescence, WB: Western blot.

### ATP release

Baseline and radiation-induced ATP release in CM was measured using a bioluminescence assay kit (Sigma-Aldrich, FLAA) and a luminometer (Tecan, Magellan software) as *per* manufacturer’s instructions. T24 cells (2 x 10^6^) in T25 flasks were treated with 50μM ARL67156 (Tocris), an ecto-ATPase inhibitor to prevent ATP hydrolysis, before irradiation at 0, 0.5, 2 and 8 Gy as described above (X-Rad 225). CM was harvested at 20 min and 60 min post-radiation for each radiation dose and was mixed with the ATP bioluminescent reagent in a 5:1 ratio. Standard curves for ATP concentration were also generated. Samples were aliquoted in white base 96-well plates before measuring the bioluminescence at 565nm. Raw values were processed to measure the ATP levels (nM) by background subtraction, normalization to cell count and the ATP standard curve.

### Western blot

Cells were cultured to sub-confluence and irradiated (2Gy). Some experiments used CM, apyrase (Sigma-Aldrich, 10U/mL, 30min) or ATP (Sigma-Aldrich, 10-100nM, 1h). Protein expression (Table 1) was detected in cell lysates by Western blotting [38].

### Live-cell imaging and Ca^2+^-fluorescence

Cells were seeded in P30 dishes (Nunc) in phenol red-free media and allowed to adhere overnight. Subsequently, cells were loaded with 1μM fluo-4AM (Invitrogen) for 30min in the incubator. The dish with loaded cells was placed under Nikon 80i upright epifluorescent microscope and washed for 15min by constant perfusion with media at room temperature (1-2ml/min) to allow dye de-esterification. Fluo-4AM was excited by a mercury lamp (Nikon) and sample bleaching reduced by neutral density filters. Appropriate filter sets were used: 465-495nm excitation, dichroic mirror 505nm and 515-555nm emission. Recordings were made using a x20 (0.75NA) lens and imaged with a Nikon DQC-FS electron multiplying charged coupled device (EMCCD) camera at 20 frames per second using WinFluor software (v3.3.4, Dr J Dempster, University of Strathclyde) and saved as.IDS files.

Changes in intracellular Ca^2+^ concentration, were reflected by changes in fluorescence intensity. A circular region of interest (ROI) was drawn around each cell in the field of view and intensity F, measured. The background-corrected ΔF was calculated by the equation ΔF=(F-F_0_)/F_0._ Background was measured by placing a ROI in an area of the dish where there were no cells. F_0_ is the mean baseline intensity of 100 frames in each recording where there was no activity. The amplitudes of transients were measured using WinFluor and summary data calculated using Microsoft Excel and Prism (v4.02, Graphpad). Statistical significance was calculated using the student’s t-test and data are expressed as mean ± standard error of the mean (SEM).

### Data analysis

Data sets were generated from three independent experiments on different days. Technical replicates in multiwall plates were averaged to produce an experimental value; experimental values were analysed and expressed as mean ± SEM. Statistical significance was determined using Student’s t-test or one-way/two-way ANOVA (Prism 4 or 5, Graphpad) and Dunnett’s multiple comparison post-hoc test with p<0.05 considered to be significant. The ATP concentration-response curve was fitted with non-linear regression (log (inhibitor) vs response, Prism) and the IC_50_ calculated.

## SUPPLEMENTARY MATERIALS FIGURES



## Abbreviations

CM: conditioned media
T24: Grade 3 urothelial cancer cells
HT1376: Grade 3 urothelial cancer cells
HUC: primary urothelial cells
SV-HUC: immortalised urothelial cells
