# Supplementary material for: Dual effects of radiation bystander signaling in urothelial cancer: purinergic-activation of apoptosis attenuates survival of urothelial cancer and normal urothelial cells
Source: Oncotarget. 2017 Oct 24;8(57):97331–43. doi: 10.18632/oncotarget.21995 (PMC5722566; doi:10.18632/oncotarget.21995)
Supplement: Supplementary file 1 [file oncotarget-08-97331-s001.pdf]

Dual effects of radiation bystander signaling in urothelial cancer: purinergic-activation of apoptosis attenuates survival of urothelial cancer and normal urothelial cells

SUPPLEMENTARY MATERIALS

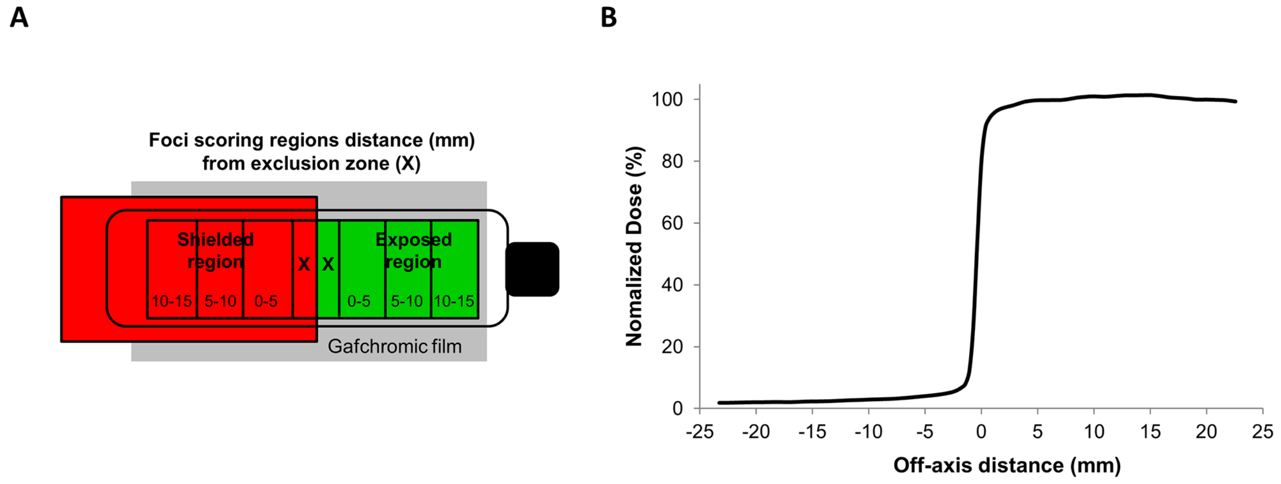

Supplementary Figure 1: Schematic diagram and dose profile for DNA DSB foci shielding experiments. (A) Cells were seeded in slide flasks with 50% of the flask shielded with MCP alloy. The flask was divided into six 5mm sections for analysis in which 50 nuclei were scored in each section. **(B)** Gafchromic EBT film was used to measure dose profiles and plotted as normalised dose against the distance on either side of the shield. An exclusion zone of 5mm was applied; foci were scored in cells outside of the exclusion zone.

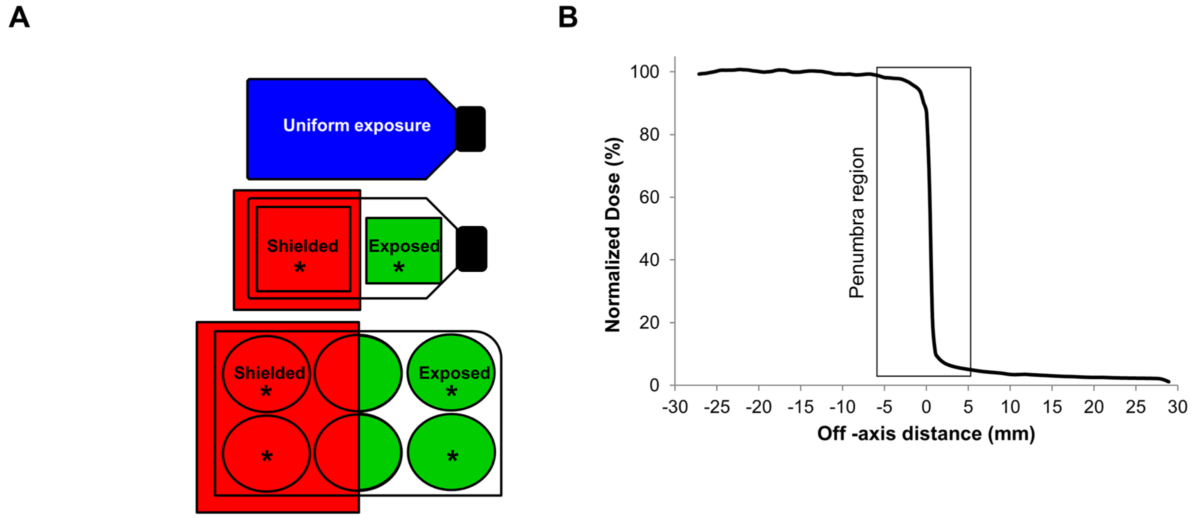

Supplementary Figure 2: Dose profiles and experimental setup for clonogenic survival shielding experiments. (A) Cells were irradiated in T25 flasks either in uniform exposures or in shielding protocols where half of the flask was shielded with MCP alloy. 5mm regions on either side of the shielding and a 5mm exclusion zone was omitted in the analysis. Analysis regions are depicted by asterisks. In experiments where communication was inhibited with a physical barrier, cells were irradiated in 6-well plates. The central wells were not analysed. **(B)** Dose profiles were measured using Gafchromic film and plotted as normalised dose against the flask distance.

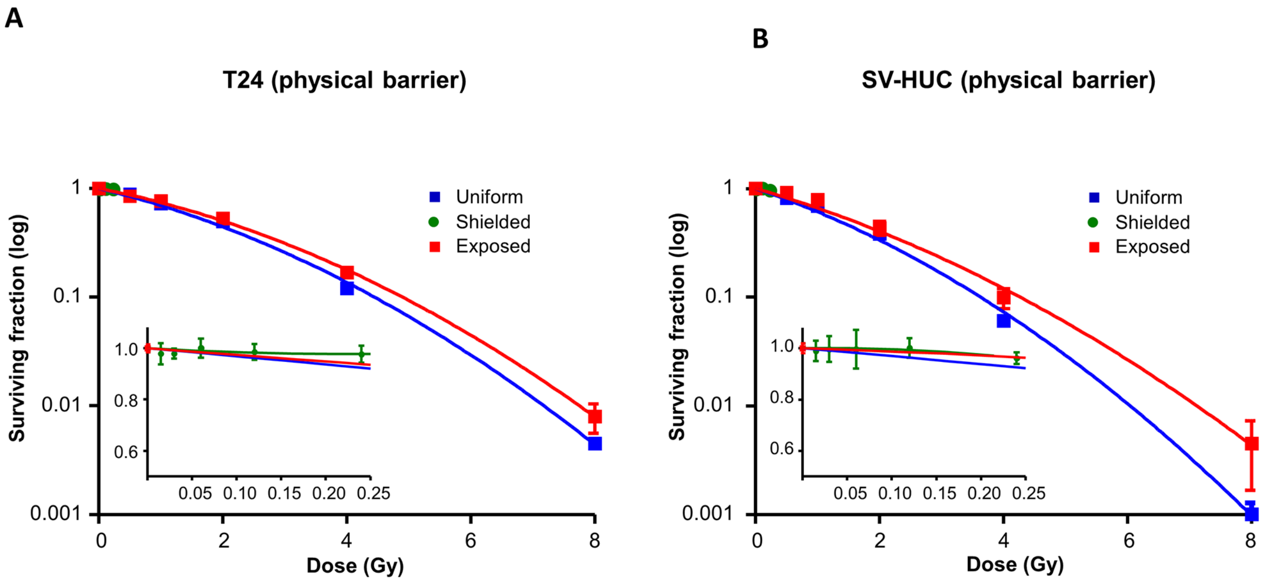

C

	T24	HT1376	SV-HUC
**Plating Efficiency**	0.49 ± 0.012	0.13 ± 0.007	0.20 ± 0.032
**α**	0.26±0.02	0.10±0.01	0.38±0.06
**β**	0.043±0.003	0.017±0.002	0.038±0.008
**α/β**	6.0±0.7	5.5±1.0	9.9±2.5

Supplementary Figure 3: Cell survival of T24 and SV-HUC after exposure to non-uniform fields where communication was physically inhibited. Responses to modulated field exposure were prevented by physical inhibition of cell-cell communication where cells were seeded in multiwell plates (N=3). Half of the plate was shielded with MCP alloy. **(A)** Cell survival response in T24 cells when exposed and shielded cells were separated by a physical barrier. ***Inset.*** The region of the graph from (A) ranging from 0-0.25Gy is shown on an enhanced scale and demonstrates the absence of a bystander effect on T24 cell survival when communication between exposed and shielded cells was inhibited. **(B)** Cell survival response in SV-HUC cells when exposed and shielded cells were separated by a physical barrier. ***Inset.*** The region of the graph from **(C)** ranging from 0-0.25Gy is shown on an enhanced scale and demonstrates the absence of a bystander effect on SVHUC cell survival when communication between exposed and shielded cells was inhibited. (C) Summary data from linear quadratic (LQ) modelling of survival curves for the T24 and SV-HUC cells in uniformly-irradiated, shielded and exposed conditions. α represents a single hit killing mechanism e.g. non-reparable DNA double stand breaks, greatest in SV-HUC whereas β represents multiple hit processes. The α/β ratio represents the dose at which both processes play an equal role.
